# Congenital internal carotid artery hypoplasia in a symptomatic young adult: A case report

**DOI:** 10.1016/j.radcr.2026.06.074

**Published:** 2026-07-06

**Authors:** Fatema Husain, Tareq AlTaei, Jenan Matar, Noor AlAnsari, Ahmet Kaya

**Affiliations:** Radiology Department, Salmaniya Medical Complex–Government Hospital, Manama, Bahrain

**Keywords:** Internal carotid artery hypoplasia, Carotid canal, Cerebral ischemia, CT angiography, Congenital vascular anomaly

## Abstract

Congenital internal carotid artery hypoplasia (CICAH) is a rare vascular anomaly, often asymptomatic due to collateral compensation, which may pose diagnostic challenges, particularly in young adults. We report the case of a 24-year-old woman who presented with headache and diplopia, in whom neuroimaging demonstrated congenital hypoplasia of the right internal carotid artery associated with chronic ischemic changes. This case highlights the uncommon symptomatic presentation of congenital internal carotid artery hypoplasia and underscores the crucial diagnostic role of radiologic imaging in identifying and differentiating it from acquired vascular disease.

## Introduction

Congenital internal carotid artery hypoplasia (CICAH) is a rare congenital vascular anomaly characterized by underdevelopment of the internal carotid artery [[1], [2], [3]]. Although early reports cited a prevalence of approximately 0.01%, modern imaging techniques have revealed a higher actual incidence by identifying previously undetected, asymptomatic patients [[1], [2], [3], [4]]. It may mimic several acquired and other congenital anomalies that may present with small-caliber ICA on vascular imaging including chronic occlusion, atherosclerotic stenosis, arterial dissection and congenital aplasia [1,4,5].

## Case presentation

A 24-year-old woman with a history of type 1 diabetes mellitus on insulin therapy had been under ophthalmology follow-up for approximately 6 months due to diplopia. At her most recent visit, she reported a 1-week history of headache in addition to persistent diplopia. Clinical examination revealed papilledema, confirmed on fundoscopy, and left abducens nerve (cranial nerve VI) palsy. No other focal neurological deficits were identified.

Routine laboratory investigations were unremarkable. Glycemic control had been well maintained, with glycated hemoglobin (HbA1c) values ranging from 34 to 41 mmol/mol, making uncontrolled diabetes a less likely primary contributor to her neurological presentation. Therefore, the patient was referred for neuroimaging for further evaluation.

Noncontrast CT of the brain demonstrated a small hypodense focus within the right frontal centrum semiovale, consistent with a chronic ischemic change (Fig. 1), with no evidence of mass effect, hydrocephalus, or other signs of raised intracranial pressure. CT angiography revealed a diffusely small-caliber petrous segment of the right internal carotid artery compared with the contralateral side (Fig. 2). No prominent collateral vessels were identified. Bone-window evaluation of the skull base demonstrated narrowing of the right carotid canal measuring approximately 3.5 mm, compared with 5.1 mm on the left (Fig. 3).

**Fig. 1:**
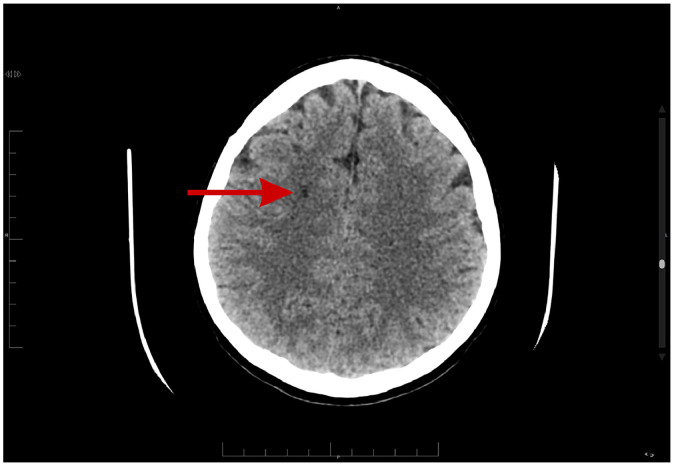
Axial noncontrast CT image demonstrating a small hypodense focus in the right frontal centrum semiovale (red arrow), consistent with chronic ischemic change.

**Fig. 2:**
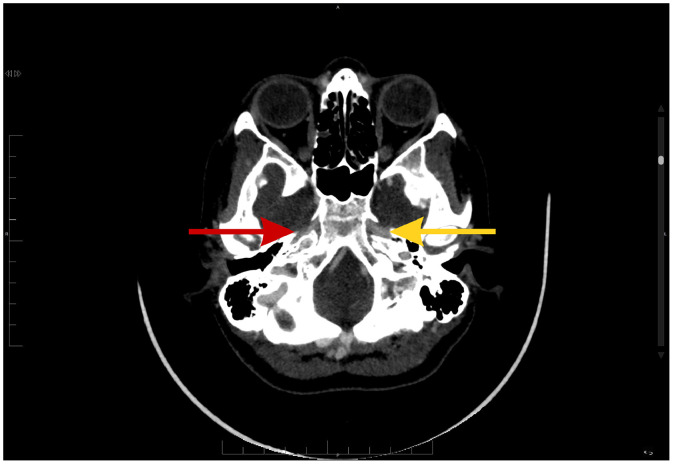
CT angiography demonstrating a diffusely small-caliber petrous segment of the right internal carotid artery (red arrow) compared with the normal caliber of the contralateral artery (yellow arrow).

**Fig. 3:**
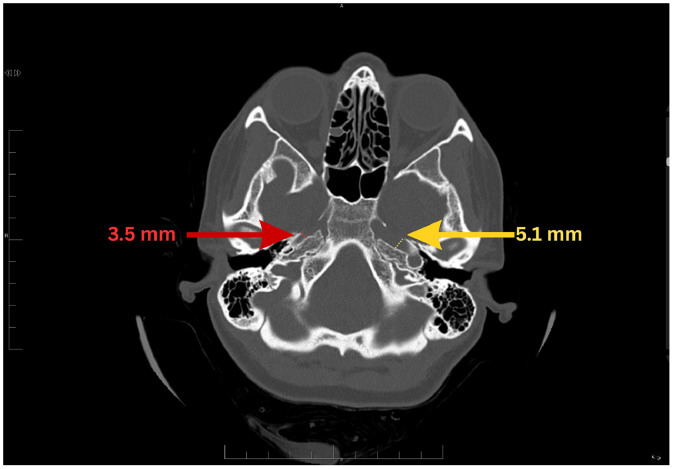
Axial bone-window CT image demonstrating narrowing of the right carotid canal (red arrow), measuring approximately 3.5 mm, compared with the normal left carotid canal (yellow arrow), which measures approximately 5.1 mm.

MRI of the brain demonstrated multiple chronic ischemic changes involving the right frontal centrum semiovale. The lesions exhibited T1-hypointense, T2/FLAIR-hyperintense signal characteristics without evidence of diffusion restriction or enhancement, confirming a diagnosis of chronic infarction with gliosis (Fig. 4, Fig. 5).

**Fig. 4:**
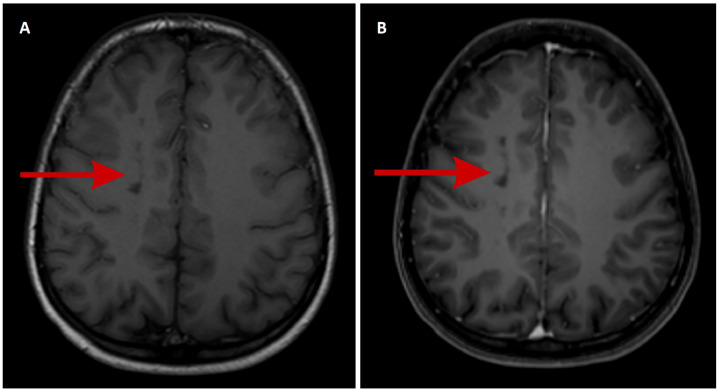
(A) Axial precontrast T1-weighted MRI image demonstrating a hypointense area within the right frontal centrum semiovale (red arrow). (B) Axial postcontrast T1-weighted MRI image demonstrating no enhancement of the lesion (red arrow).

**Fig. 5:**
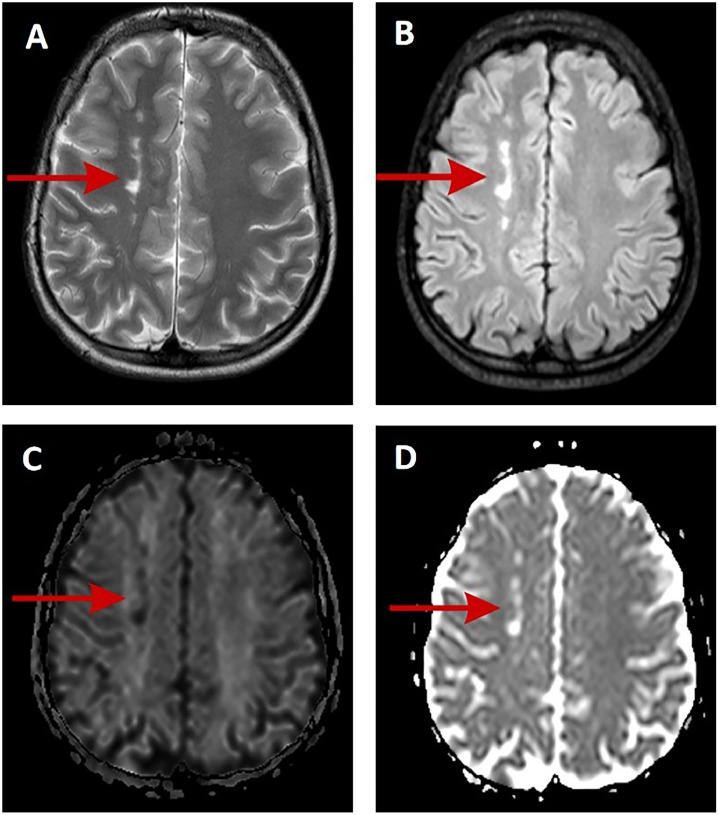
(A) Axial T2-weighted MRI image demonstrating hyperintense signal in the right frontal centrum semiovale (red arrow). (B) Axial FLAIR image demonstrating corresponding hyperintense signal in the same region (red arrow). (C) Axial diffusion-weighted imaging (DWI) demonstrating no diffusion restriction (red arrow). (D) Apparent diffusion coefficient (ADC) map demonstrating no corresponding low signal (red arrow).

The patient was started on dual antiplatelet therapy for secondary ischemic prevention. During hospitalization, her headache improved, with partial resolution of diplopia. No new neurological deficits developed. She was discharged in stable condition with instructions for outpatient follow-up and interval MRI surveillance. However, the patient did not attend subsequent follow-up appointments, and long-term clinical and imaging follow-up data were therefore unavailable.

## Discussion

Congenital internal carotid artery hypoplasia (CICAH) results from incomplete embryologic development of the internal carotid artery and is characterized by diffuse arterial narrowing, typically beginning 1-2 cm above the carotid bifurcation [[1], [2], [3]].

When symptomatic, patients most commonly present with ischemic events such as stroke or transient ischemic attack [1,5,6]. Less frequent manifestations include headache, cranial nerve palsy, visual disturbance, and seizures [[1], [2], [3], [4],^5^]. Rarely, subarachnoid hemorrhage secondary to aneurysm rupture has been reported [1,3]. Due to its nonspecific clinical presentation, CICAH may be overlooked or misdiagnosed, particularly in adult patients undergoing imaging for vague neurological symptoms [1,4]. Consequently, accurate diagnosis relies predominantly on characteristic radiological features rather than clinical findings alone [1,4].

The patient’s acute presentation with headache prompted further imaging evaluation and may represent a symptomatic manifestation of congenital internal carotid artery hypoplasia, as headache has been reported among the recognized clinical presentations of this condition [[1], [2], [3], [4], [5]]. Her chronic left abducens nerve palsy was considered less likely to be related to congenital internal carotid artery hypoplasia and was instead presumed to be secondary to diabetic microangiopathy, given its chronic course, contralateral location relative to the hypoplastic right internal carotid artery, and the absence of imaging findings supporting a direct compressive, ischemic, or aneurysmal mechanism. The right-sided watershed distribution of the chronic cerebral infarction identified on CT, in association with narrowing of the right carotid canal and ipsilateral internal carotid artery hypoplasia, strongly supported congenital internal carotid artery hypoplasia as the primary underlying vascular etiology of the patient’s cerebral infarction.

Differentiating CICAH from other causes of internal carotid artery narrowing is important because it guides clinical management and determines whether vascular intervention may be warranted. The differential diagnosis of CICAH includes several congenital and acquired conditions that may present with a small-caliber internal carotid artery on vascular imaging. Therefore, evaluation of the carotid canal on skull-base CT remains crucial for distinguishing congenital from acquired etiologies [1]. Differential diagnosis include ICA aplasia which is a more severe congenital anomaly that can mimic CICAH and is characterized by complete absence of the ICA and, importantly, absence of the ipsilateral carotid canal [1,3,5]. Another differential diagnosis is chronic ICA occlusion that may demonstrate a markedly narrowed or absent arterial lumen, however, the carotid canal is typically normal in size because osseous development occurs before the acquired vascular insult [1,4,7]. Similarly, ICA near-occlusion caused by severe atherosclerotic stenosis may produce distal arterial collapse and apparent hypoplasia, but is usually associated with a focal high-grade stenotic segment and a normally developed carotid canal [1,7]. ICA dissection can also mimic hypoplasia due to long-segment luminal narrowing, although characteristic imaging findings such as an intimal flap, mural hematoma, tapered “flame-shaped” stenosis, or pseudoaneurysm favor an acquired dissection rather than a congenital anomaly [4].

Imaging plays a crucial role in distinguishing congenital internal carotid artery hypoplasia from acquired vascular narrowing, particularly evaluation of the bony carotid canal [1]. The internal carotid artery (ICA) originates during embryonic development from the first and third aortic arches, as well as the dorsal aorta [1,2]. Between the fourth and sixth weeks of gestation, the ICA develops concurrently with the carotid canal [3]. Consequently, evaluation of the carotid canal can aid in distinguishing congenital ICA agenesis from acquired internal carotid artery stenosis (ICAS). The absence or narrowing of the carotid canal suggests a congenital etiology [4]. This developmental relationship explains the association between arterial hypoplasia and carotid canal narrowing, although canal narrowing alone is not absolutely pathognomonic [1,4]. Therefore, additional supporting findings, including the absence of intracranial atherosclerotic or microangiopathic changes on neuroimaging, favor a congenital over an acquired pathology [1,4].

While CICAH is typically asymptomatic due to Circle of Willis collateralization [[1], [2], [3], [4]], our patient lacked prominent collateral vessels on CTA. Evaluation of the Circle of Willis did not demonstrate dominant collateralization through the anterior or posterior communicating arteries, and no associated intracranial aneurysm or additional congenital cerebrovascular anomaly was identified. However, her survival into young adulthood without prior major stroke suggests reliance on microscopic leptomeningeal or transdural anastomoses below CTA resolution. It is possible that failure of these fragile networks, potentially exacerbated by diabetic microangiopathy, contributed to the observed ischemic changes. Importantly, these ischemic changes were strictly unilateral and confined to the watershed territory of the hypoplastic artery. This focal distribution distinguishes the pathology from primary diabetic small vessel disease, which typically manifests as diffuse, bilateral white matter changes [[8], [9], [10], [11], [12]], thereby supporting the congenital anomaly as the primary etiology.

Carotid duplex ultrasound is a noninvasive first-line modality for evaluating the extracranial internal carotid artery when acquired carotid stenosis is suspected [4,7]. In congenital internal carotid artery hypoplasia, ultrasound may demonstrate a uniformly small-caliber artery with globally reduced flow velocities rather than focal stenosis [4,7,13]. However, these findings are not specific and may overlap with other vascular conditions, limiting the ability of ultrasound to reliably differentiate congenital hypoplasia from acquired pathology [4,7,13]. Therefore, further evaluation with cross-sectional imaging is often required for definitive diagnosis.

In this patient, carotid duplex ultrasound was not performed because the acute presentation with headache and papilledema necessitated urgent cross-sectional neuroimaging to exclude elevated intracranial pressure and intracranial pathology.

CT and CT angiography allow simultaneous assessment of brain parenchyma, arterial anatomy, and skull-base structures [1,3]. Noncontrast CT is useful for identifying parenchymal ischemic sequelae and excluding acute intracranial pathology, while CT angiography enables detailed evaluation of arterial caliber and course. Associated osseous anatomy involving the carotid canal can be assessed using bone-window.

In this patient, noncontrast CT demonstrated chronic unilateral ischemic changes, prompting vascular evaluation with CT angiography. CTA was pivotal in establishing the diagnosis by demonstrating diffuse hypoplasia of the right internal carotid artery with corresponding narrowing of the ipsilateral carotid canal.

MRI provides superior evaluation of parenchymal and hemodynamic consequences [[10], [11], [12]]. MRI is particularly useful for assessing the distribution of white-matter changes, supporting differentiation between localized, territory-based abnormalities and diffuse bilateral involvement typically associated with cerebral small-vessel disease [8,9]. In this case, MRI findings supported a congenital etiology by demonstrating chronic ischemic changes confined to the vascular territory of the hypoplastic internal carotid artery.

Digital subtraction angiography remains the gold standard for detailed assessment of cerebral vasculature [1,3]. Due to its invasive nature, it was not undertaken in this case and is usually reserved for situations in which noninvasive imaging is inconclusive or when endovascular intervention is being considered [1,6].

Management of CICAH is generally conservative [1]. Treatment focuses on lifestyle modification along with antiplatelet therapy for secondary prevention in patients with ischemic manifestations [1,6]. Surgical or endovascular intervention is reserved for selected cases, particularly when associated intracranial aneurysms, significant hemodynamic compromise, or progressive ischemia are present [3]. Routine clinical and imaging follow-up is recommended, as ICAH remains an under-recognized cause of stroke [[1], [2], [3]].

## Conclusion

Congenital hypoplasia of the internal carotid artery is a rare vascular anomaly that may mimic acquired occlusive disease. Although most cases are asymptomatic, a subset of patients may present with nonspecific neurological or ischemic symptoms. Accurate diagnosis relies on careful radiologic evaluation, particularly cross-sectional imaging with angiographic assessment and exclusion of acquired causes.

## Declaration of generative AI and AI-assisted technologies in the writing process

A generative AI tool (ChatGPT, Gemini) was used to assist in language editing and improving clarity of the manuscript. The authors reviewed, edited, and approved the final version and take full responsibility for the content.

## Ethical approval

Ethical approval was not required for this case report.

## Patient consent

Written informed consent was obtained from the patient for publication of this case report and accompanying images.
